# General Protocol to Obtain D‐Glucosamine from Biomass Residues: Shrimp Shells, Cicada Sloughs and Cockroaches

**DOI:** 10.1002/gch2.201800046

**Published:** 2018-08-24

**Authors:** Diego L. Bertuzzi, Tiago B. Becher, Naylil M. R. Capreti, Julio Amorim, Igor D. Jurberg, Jackson D. Megiatto, Catia Ornelas

**Affiliations:** ^1^ Institute of Chemistry University of Campinas – Unicamp Campinas SP 13083‐970 Brazil

**Keywords:** biomass, chitin, glucosamine, mutarotation, residues

## Abstract

A general protocol is developed to obtain D‐glucosamine from three widely available biomass residues: shrimp shells, cicada sloughs, and cockroaches. The protocol includes three steps: (1) demineralization, (2) deproteinization, and (3) chitin hydrolysis. This simple, general protocol opens the door to obtain an invaluable nitrogen‐containing compound from three biomass residues, and it can potentially be applied to other chitin sources. White needle‐like crystals of pure D‐glucosamine are obtained in all cases upon purification by crystallization. Characterization data (NMR, IR, and mass spectrometry) of D‐glucosamine obtained from the three chitin sources are similar and confirm its high purity. NMR investigation demonstrates that D‐glucosamine is obtained mainly as the α‐anomer, which undergoes mutarotation in aqueous solution achieving equilibrium after 440 min, in which the anomeric glucosamine distribution is 60% α‐anomer and 40% β‐anomer.

## 1 Introduction

Nitrogen atoms play a fundamental role in the process of storage and transfer of genetic information as they are essential for the synthesis and assembly of DNA and RNA. Nitrogen is also required to make amino acids that are the building blocks of proteins, the essential catalysts, and structural components of all plant and animal cells.^1^, ^2^, ^3^ Nitrogen is an abundant element on Earth as our atmosphere is composed of about 80% nitrogen. Paradoxically, this large quantity of nitrogen is in the form of diatomic nitrogen, a nearly chemical inert species to which the vast majority of living things are biologically indifferent. The inertness of nitrogen gas comes from the unique chemistry of the nitrogen element, in which there is a large difference in binding energy between nitrogen atoms linked to each other by single bonds (−80 kJ mol^−1^) or to other atoms such as carbon (−308 kJ mol^−1^ for single N—C bonds), hydrogen (−391 kJ mol^−1^), and oxygen (−201 kJ mol^−1^ for single N—O and −607 kJ mol^−1^ for N=O bonds) versus the N≡N triple bond (−945 kJ mol^−1^), which renders the latter one of the strongest chemical bonds known.^4^, ^5^

At high temperatures, the bond between the two nitrogen atoms breaks to produce reactive nitrogen species that combine with other elements, such as oxygen and hydrogen, to form N‐containing compounds that can be absorbed by living organisms. In the atmosphere, lightning and meteor trails are hot enough to produce measurable amounts of biologically useful N‐containing compounds that are eventually brought down to living organisms by rain.^6^, ^7^ Another natural way to provide life with usable N‐containing compounds is the fixation process, which is promoted by a few genera of bacteria and blue‐green algae (cyanobacteria). Cyanobacteria are equipped with “nitrogenase” that is composed of two proteins bearing iron and molybdenum‐iron cofactors able to chemically activate and convert nitrogen gas into ammonia (NH_3_) and ammonium ion (NH_4_^+^) at mild temperatures. NH_3_ and NH_4_^+^ along with nitrites (NO_2_^−^) and nitrates (NO_3_^−^), which are produced from nitrification reactions (oxidation of ammonia by oxygen promoted by bacteria of the genus *Nitrosomonas* and *Nitrobacter*, respectively) are water soluble and reactive species that can be more easily assimilated by biological systems.^8^, ^9^, ^10^, ^11^, ^12^ No higher or multicellular organisms have developed the capability to fix their own nitrogen, although some participate indirectly by forming symbiotic associations with nitrogen‐fixing bacteria. Even though fixing processes provide organisms with biological useful nitrogen, life demands high levels of it to flourish. Therefore, nitrogen is a scarce nutritional resource and is typically the limiting factor to living organisms' growth.^3^, ^8^

Since the invention of agriculture, humans have realized that harvesting continuously removes “fixed” nitrogen from the soil. Therefore, nitrogen has had to be supplied somehow to the soil to keep up with food demand. The first nitrogen source used by humans to enrich the soil was manure, followed by guano (pigeon's droppings) and saltpeter (chiefly composed of sodium and potassium nitrate) extracted from natural reservoirs in the Pacific Islands and in the deserts of Chile, respectively. However, it was not until the development of the first industrial process to convert nitrogen gas from the air into ammonia by the German scientists Fritz Haber and Carl Bosch in the 1900s that nitrogen fertilizers became readily available in large scale for food production.^3^, ^13^ The Haber–Bosch ammonia process is very efficient, affording ammonia in yields close to the theoretical value. To achieve that efficiency, the process requires nitrogen and hydrogen gases compressed under extremely high pressures of several hundreds of atmospheres and elevated temperatures (about 500 °C). Fossil fuels provide the process with both the energy necessary to achieve such high temperature and pressures, and the starting material hydrogen gas, which is afforded from the steam reforming reaction of natural gas. Production of hydrogen gas consumes between 3% and 5% of the world's natural gas production whereas the Haber–Bosch process consumes about 2% of the total global energy output. In other words, to make ammonia from nitrogen gas requires a lot of energy. Traditionally, that energy has been afforded from burning fossil fuels, which in turn has produced large amounts of carbon dioxide and has significantly contributed to the increase of the green‐house effect on the planet.^14^

**Figure 1:**
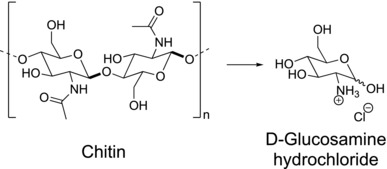
Chitin hydrolysis to obtain D‐glucosamine hydrochloride.

Given such challenges to provide life with biological useful nitrogen, it is crucial to find natural and renewable sources of “fixed” nitrogen.^15^, ^16^, ^17^ In the past decades, production of useful chemical compounds from biomass residues has grown in importance due to the high consumption of Earth's natural resources.^18^, ^19^ Chitin, a nitrogen‐containing polymer, is the second most abundant natural polymer on Earth, being cellulose the first one. Chitin is a linear biomacromolecule composed of *N*‐acetyl glucosamine subunits linked together through covalent β(1 → 4) glycosidic bonds (**Figure** **1**).^20^ In the biosphere, ≈10^12^ – 10^14^ tons of chitin are produced every year by several organisms, ranging from fungi and some algae to arthropods, the largest phylum of animals on the planet, both in the number of species and total number of individuals that include all insects, arachnids, and crustaceans.^20^, ^21^

The industrial processing of crustaceans for food production generates large amounts of waste, in which 50%–60% of the total dry weight is shells.^19^ Such composite structure has low biodegradation rates^22^, ^23^, ^24^; therefore, accumulation of large quantities of shells has become a major concern.^25^, ^26^ Global production of crustacean shells is estimated to be about 6–8 million tons a year, from which 15%–40% in weight is chitin.^15^, ^16^, ^27^, ^28^ A minor fraction of this biopolymer is extracted and used in several applications such as flocculants in water treatment, textiles, plastic products, as well as in cosmetic and biomedicine formulations.^29^ However, the major part of the crustacean shells containing the invaluable chitin is discarded in landfills.

Several processes for chitin extraction have been developed, based on chemical^30^, ^31^, ^32^, ^33^, ^34^ or microorganism‐mediated fermentation processes.^22^, ^35^, ^36^ Although more aligned with the principles of green chemistry, bioextraction methods have not yet achieved commercialization, due to their high costs.^22^ Several studies report the optimization of the chemical extraction of chitin or chitosan from crustacean sources.^32^, ^37^, ^38^, ^39^ Recently, Chen et al. have reported a solvent‐free deacetylation method based on ball milling a mixture of pure chitin and sodium hydroxide, which provided low disperse chitosan.^40^

Acidic hydrolysis of chitin (Figure 1) promotes both the cleavage of the glycosidic bond and the deacetylation reaction to yield D‐glucosamine (2‐amino‐2‐desoxy‐D‐glucose).^30^, ^31^ This amino sugar can be found in the human body, acting as a precursor to glycosaminoglycans in cartilage, and has an important role in the formation of joints, sinews, bones, heart valves, and respiratory tract. Therefore, D‐glucosamine and its salts are employed in dietary supplement products, which have demonstrated positive effects on the treatment of osteoarthritis, knee, and back pain.^41^, ^42^, ^43^ In the context of sustainable chemistry, D‐glucosamine was recently proposed as a renewable precursor for the preparation of valuable specialty chemicals, such as 5‐(hydroxymethyl)furfural and other furfural derivatives.^17^, ^44^, ^45^

The use of co‐solvents during the acidic hydrolysis has been proposed as a strategy to decrease the concentration of corrosive acids used during chitin hydrolysis. Recently, Zhang and Yan demonstrated that using diethylene glycol diethyl ether as co‐solvent provided glucosamine in 80% yield of from pure chitin using low concentration of sulfuric acid at 175 °C, for 1 h.^46^

Having in mind the importance of obtaining D‐glucosamine from renewable sources, this work describes the isolation of pure D‐glucosamine from three biomass residues: shrimp shells, cicada sloughs, and cockroaches, using a general protocol that works for all chitin sources tested.

## 2 Results and Discussion

**Figure 2:**
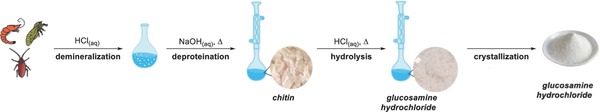
Schematic representation of the general procedure to obtain D‐glucosamine from shrimp shells, cicada sloughs, and cockroaches.

Several studies have demonstrated that D‐glucosamine can be obtained from shrimp shells using three main steps: demineralization, deproteinization, and chitin hydrolysis.^32^, ^37^, ^38^, ^47^ Here we demonstrate that these three steps can be used as an optimized protocol to obtain crystalline D‐glucosamine from three biomass residues: (a) shrimp shells, (b) cicada sloughs, and (c) cockroaches (**Figure** **2**).

Shrimp shells from Whiteleg shrimp (*Litopenaeus vannamei)* were collected from local markets in the state of Sao Paulo, Brazil. The definition of shrimp shells is understood here as shrimps' abdominal segments, carapace, antennae, and tail. Chemical composition of shrimp shells includes 15%–25% chitin, 30%–40% minerals, 30%–50% proteins and pigments, and 0.3%–1% lipids.^26^ The shells are kept in closed plastic bags in the freezer until 2 h prior to the experiment. The shells are thoroughly washed in tap water to remove dirt and excess of loose tissues, and once with distilled water prior to drying.

Cicadas can be found in regions with temperate to tropical climates. Cicadas molt their cuticles leaving their sloughs behind attached on trees. Cicada sloughs consist of ≈37% chitin, 40% proteins, 12% minerals, 3% lipids, and 8% water.^48^ Sloughs from Giant cicadas (*Quesada gigas*) were collected from trees located at the University of Campinas campus (Campinas, São Paulo, Brazil) during October 2015 and October 2016 (Spring), and used without any previous treatment.

Cockroaches can be found throughout the world as the species can withstand a wide range of environments, from hot subtropical deserts to the extremely cold temperatures of the Artic. Due to their global availability and abundance, cockroaches are an interesting source of chitin as their exoskeleton and wings contain about 13%–18% of the biopolymer. Nymph and adult American cockroaches (*Periplaneta americana*) were handpicked in random locations in Campinas, São Paulo, Brazil, and used with no previous treatment.

### 2.1 Demineralization

In arthropods' exoskeleton, biominerals provide mechanical strength and calcium storage.^49^, ^50^ These biominerals consist of calcium carbonate, varying amounts of crystalline calcite, small amounts of calcium phosphate, minerals based on Na, K, Mg, Sr, and traces of minerals based on Fe, Cu, Ni, and Ba.^26^, ^51^, ^52^ To remove these minerals, the chitin source is stirred for 3 h, in HCl 0.5 m, at room temperature. The calcium carbonate present in the exoskeleton reacts with HCl resulting in soluble CaCl_2_, while releasing CO_2_. After removing the minerals, for all three chitin sources one observes the loss of stiffness of what previously were rigid exoskeletons. After demineralization, the exoskeletons are washed with distilled water and submitted to deproteinization.

### 2.2 Deproteinization

Proteins, pigments, and lipids are removed by treating the previously demineralized chitin source with 1.0 m aqueous NaOH, at reflux, for 2 h. Combination of high pH and high temperature promotes denaturation and partial hydrolysis of proteins, thereby rendering them water soluble. During reflux, formation of foam inside the round‐bottom flask suggested the saponification of lipids. Solubilization of pigments afforded solutions with distinct color tones according to the chitin source: orange for shrimp shells, dark brown for cicada sloughs, and black for cockroaches (see the Supporting Information for photos).^53^ After demineralization and deproteinization, nearly pure off‐white chitin was afforded when shrimp shells were used as chitin source whereas the chitin obtained from cicada sloughs and cockroaches presented a dark‐brown coloration. This observation suggests that the pigments present in the shrimp shells (mainly Astaxanthin) are solubilized during the deproteinization process being easily separated from chitin. However, the pigments present in the cicada sloughs and cockroaches (predominantly insoluble melanin derivatives) are not easily separated from chitin during this step. To obtain pure D‐glucosamine from impure chitin, the remaining pigments and other impurities were eliminated during the crystallization process.

### 2.3 Chitin Hydrolysis

Chitin hydrolysis is carried out with concentrated HCl (37%) at 90 °C, for 2 h. Two distinct hydrolysis events occur during this step (Figure 1): i) cleavage of the glycosidic bonds, depolymerizing chitin into its monomeric units, and ii) cleavage of the amide bonds that lead to deacetylation of the glucosamine subunits. Crystallization using ethanol/water affords white needle‐like crystals of pure D‐glucosamine hydrochloride, which are filtered off and dried under vacuum.

Chitin content on the three biomass sources as well as the average yields of the obtained glucosamine is gathered in **Table** **1**. For each chitin source three independent experiments were carried out in order to demonstrate the protocol reproducibility and to calculate an average yield. The highest yields of glucosamine were obtained from cicada sloughs, due to the initial higher content of chitin in this biomass residue.

**Table 1:** Yields obtained for synthesis of pure D‐glucosamine from chitin extracted from shrimp shells, cicada sloughs, and cockroaches

Chitin source	Chitin content (literature)	Glucosamine yield % (w/w)b)	Glucosamine average yield % (w/w)
Shrimp shells	15%–25%^26^	16.68%	13.70%
		12.65%	
		11.77%	
Cicada sloughs	37%^48^	20.82%	20.46%
		20.62%	
		19.94%	
Cockroaches	15%–20%a), 54	3.43%	3.28%
		3.33%	
		3.09%	

^a)^ This value refers to the chitin content in the cockroaches' wings, but in this experiment whole cockroaches were used

^b)^ Three independent experiments were carried out for each chitin source; yield = (mass of glucosamine obtained/mass of initial biomass residue) × 100%.

### 2.4 Characterization of Glucosamine Obtained from Shrimp Shells, Cicada Sloughs, and Cockroaches

Glucosamine obtained from the three biomass residues was characterized by NMR (^1^H, ^13^C, COSY), IR, and mass spectrometry. As expected, characterization data for glucosamine obtained from the three chitin sources are similar.

**Figure 3:**
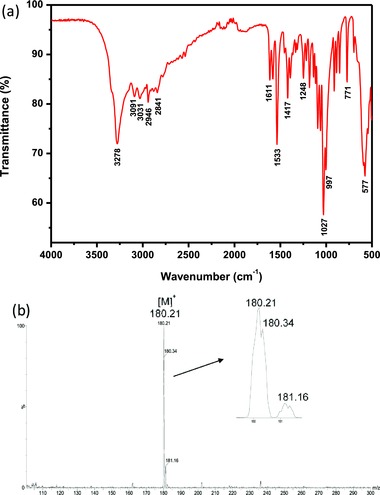
a) IR and b) mass spectra of D‐glucosamine obtained from shrimp shells; similar spectra were obtained for the glucosamine isolated from cicada sloughs and cockroaches.

IR spectrum confirms complete deacetylation of glucosamine by showing the typical bands attributed to the vibrating modes of the OH and NH bonds in the ≈2500–3500 cm^−1^ region, as well as the absence of the C=O stretching band at ≈1700 cm^−1^ (**Figure** **3**a).^55^ Mass spectrometry reveals a single peak at *m/z* 180.21 [M‐Cl]^+^, which corresponds to the molecular ion mass for D‐glucosamine (*m/z* 215.06, calculated for C_6_H_14_NO_5_Cl) with the expected isotopic distribution (Figure 3b).

**Figure 4:**
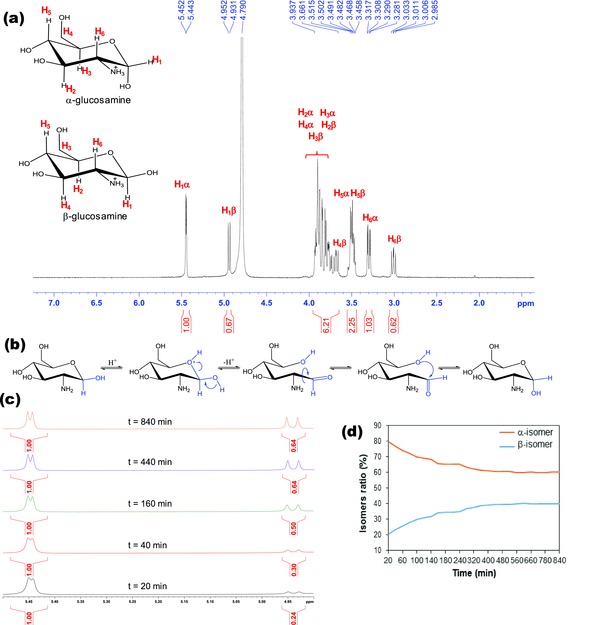
^1^H NMR data (400 MHz, 298 K) of glucosamine hydrochloride in D_2_O: a) ^1^H NMR spectrum with anomeric distribution at equilibrium; b) mutarotation mechanism of glucosamine; c) anomeric region of the ^1^H NMR spectra recorded at different elapsed times; and d) relative amount of each anomer as a function of time.

The ^1^H NMR spectrum of D‐glucosamine shows two signals at 5.45 and 4.94 parts per million (ppm) that are attributed to the proton linked to the anomeric carbon (**Figure** **4**a). These signals are the most downfield shifted, because the anomeric carbon is deshielded by the two adjacent oxygen atoms.^56^ According to the Karplus relationship, the *J*‐coupling constants depends on the dihedral angle between the two coupling protons.^57^, ^58^ For axial‐equatorial coupling (α‐anomer, θ ≈ 60 °C) *J*‐coupling values vary between 1 and 7 Hz whereas for trans diaxial coupling (β‐anomer, θ ≈ 180 °C) *J*‐coupling values vary between 8 and 14 Hz.^59^ The doublet at 5.45 ppm has *J* = 3.6 Hz while the peak at 4.94 ppm has *J* = 8.4 Hz. Therefore, the two doublets at 5.45 and 4.94 ppm correspond to the protons linked to the anomeric carbon in α and β‐anomers, respectively. The other nuclei on the glucosamine hydrochloride structure resonate at the expected chemical shifts and multiplicity (Figure 4a).^56^

The α and β‐anomers of glucosamine are stable solids; however, in aqueous solution they undergo interconversion to reach an equilibrium distribution (mutarotation). Mutarotation occurs via ring opening after protonation of the ring oxygen affording the aldehyde acyclic intermediate. The carbonyl group in the open structure can suffer conformational change through rotation of the C—CO bond, resulting in conversion of anomers after ring closing.^60^ The glucosamine mutarotation process was investigated in aqueous solution by acquiring ^1^H NMR spectra at distinct elapsed times after solubilization in D_2_O (Figure 4c; see the Supporting Information for complete ^1^H NMR spectra of D‐glucosamine hydrochloride at all time points recorded). Data show that at the first time point (20 min) glucosamine existed as 80% α‐anomer and 20% β‐anomer. As time elapsed, the amount of β‐anomer increased at the expense of the α‐anomer. The mutarotation process reached equilibrium at *t* = 440 min, in which the anomeric glucosamine distribution was about 60% α‐anomer and 40% β‐anomer (Figure 4b–d).

## 3 Conclusion

A general protocol was optimized to obtain the invaluable nitrogen‐containing compound D‐glucosamine from three widely available biomass residues: shrimp shells, cicada sloughs, and cockroaches. The protocol includes three steps: (1) demineralization, (2) deproteinization, and (3) chitin hydrolysis. Upon purification by crystallization, white needle‐like crystals of pure glucosamine were obtained in all cases. NMR, IR, and MS data of glucosamine obtained from the three chitin sources are similar and demonstrate its high purity. This process does not obey all the principles of Green Chemistry because it uses corrosive HCl and NaOH. However, when analyzing the green metrics of this organic synthesis protocol several advantages can be highlighted^61^: (i) the starting materials are biomass residues widely available worldwide, (ii) it uses inexpensive substrates, (iii) it uses only water as solvent in all steps, (iv) high carbon efficiency (75%), (v) high atom utilization, and high atom economy. This simple general protocol opens the door to obtain an invaluable nitrogen‐containing compound from three biomass residues, and it can potentially be applied to other biomass chitin sources.

## 4 Experimental Section

*NMR*: ^1^H and COSY NMR spectra were recorded at 400.0 MHz and 25 °C on a Bruker AC 400 spectrometer. ^13^C NMR spectra were acquired at 100.0 MHz and 25 °C on a Bruker AC 400 spectrometer. All chemical shifts are reported in ppm relative to the solvent residual peak.

*Mass Spectrometry*: The mass spectrum was obtained using a Waters Micromass Quattro Micro API instrument using electrospray ionization and triple quadrupole mass analyzer. The sample was analyzed by direct infusion with methanol/water/formic acid 49.9:50:0.1 (v/v). Experimental conditions: Capillary voltage: 3 kV; cone voltage: 15 V; extractor voltage: 3 V; radio frequency lens: 0.5 V; source temperature: 150 °C; desolvation temperature: 200 °C; desolvation gas flow: 800 L h^−1^; and cone gas flow: 50 L h^−1^.

*Infrared Spectroscopy*: Infrared spectra were recorded using an Agilent Cary 630 Fourier‐transform infrared spectroscopy (FTIR) spectrometer with the attenuated total reflectance accessory.

*General Procedure for Demineralization*: The chitin source was stirred in 700 mL of the aqueous HCl 0.5 m solution at room temperature for 3 h. The solution was filtered and the solid was washed thoroughly with distilled water, and dried (see the Supporting Information for detailed experimental procedures).

*General Procedure for Deproteinization*: The demineralized solid was introduced in a 1 L round‐bottom flask. 400 mL of a NaOH 1.0 m aqueous solution was added, and the reaction mixture was submitted to reflux during 2 h. The solution was filtered, and the obtained chitin was washed with distilled water, and dried (see the Supporting Information for detailed experimental procedures).

*General Procedure for Chitin Hydrolysis to Obtain D‐Glucosamine Hydrochloride*: Chitin was introduced in a 500 mL round‐bottom flask, and 80 mL of concentrated HCl (37%) was added. Under stirring, the solution was heated at 90 °C for 2 h. In this step, chitin dissolved and the solution color became brownish. The solution was cooled to 60 °C and 10 mL of distilled water was added. Activated charcoal was added, and the solution was stirred at 60 °C for 30 min. The solution was filtered twice through paper. Ethanol was added and the solution was kept in the fridge to allow crystallization of glucosamine hydrochloride. The white crystals were filtered, washed with cold ethanol, and dried under high vacuum (see the Supporting Information for detailed experimental procedures).

*^1^H NMR (400 MHz, D_2_O, δ_ppm_)*: 5.46 (d, *J* = 3.6 Hz, 1H), 4.96 (d, *J* = 8.4 Hz, 1H), 3.94–3.68 (m, 8H), 3.55–3.47 (m, 2H), 3.31 (dd, *J* = 10.6 and 3.6 Hz, 1H), 3.02 (dd, *J* = 10.6 and 8.4 Hz, 1H). ^13^C NMR (100 MHz, D_2_O, δ_ppm_): 92.7, 89.2, 76.2, 72.0, 71.6, 69.7, 69.6, 60.5, 60.3, 56.7, 54. ESI‐MS (M)^+^ *m/z* calcd for C_6_H_14_NO_5_^+^: 180.09, found 180.21. FTIR (cm^−1^): 3346, 3278, 3091, 3031, 2841, 1611, 1583, 1533, 1417, 1387, 1248, 1181, 1092, 1027, 997, 914, 882, 851, 771, 577, 544, 498.

## Conflict of Interest

The authors declare no conflict of interest.

## Supporting information

Supplementary
