# Supplementary material for: General Protocol to Obtain D‐Glucosamine from Biomass Residues: Shrimp Shells, Cicada Sloughs and Cockroaches
Source: Glob Chall. 2018 Aug 24;2(11):1800046. doi: 10.1002/gch2.201800046 (PMC6607258; doi:10.1002/gch2.201800046)
Supplement: Supplementary file 1 — Supplementary [file GCH2-2-1800046-s001.pdf]

Copyright WILEY-VCH Verlag GmbH & Co. KGaA, 69469 Weinheim, Germany, 2018.

Supporting Information

for *Global Challenges,* DOI: 10.1002/gch2.201800046

General Protocol to Obtain D-Glucosamine from Biomass Residues: Shrimp Shells, Cicada Sloughs and Cockroaches

*Diego L. Bertuzzi, Tiago B. Becher, Naylil M. R. Capreti, Julio Amorim, Igor D. Jurberg, Jackson D. Megiatto Jr., and Catia Ornelas**

General Protocol to Obtain D-Glucosamine From Biomass Residues: Shrimp Shells, Cicada Sloughs and Cockroaches

Diego L. Bertuzzi, Tiago B. Becher, Naylil M. R. Capreti, Julio Amorim, Igor D. Jurberg, Jackson D. Megiatto Jr., Catia Ornelas*

Institute of Chemistry, University of Campinas- Unicamp, Campinas, SP 13083-970, Brazil

catiaornelas@catiaornelaslab.com

Supporting Information

1. Glucosamine Hydrochloride from shrimp shells

Shrimp shells. Shells from Whiteleg shrimp (*Litopenaeus vannamei)* were collected from local markets. The definition of shrimp shells is understood here as the shrimps' abdominal segments, carapace, antennae and tail. Usually the shells came along with significant amount of water/ice and loose tissues. The shells are kept in closed plastic bags in the freezer until 2h prior to the experiment. Weigh about 200g of wet shells in a glass bowl. Thoroughly wash the shells with tap water to remove dirt and excess of loose tissues, and once with distilled water. The shells were dried by pressing them against several layers of paper and the operation was repeated until the paper came out dry. After washing and drying, 100 g of shells were weighted and used.

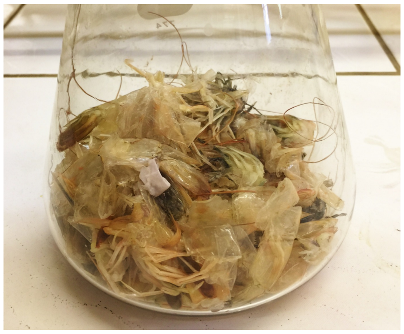

Figure S1. Picture of the shrimp shells obtained after washing and drying.

1.1. Experimental procedure for demineralization of shrimp shells. Introduce 100 g of dry shrimp shells in a 1L Erlenmeyer and add 700 mL of an aqueous solution of HCl 0.5. Stir the suspension at room temperature for 3h. Filter the solution using a plastic sieve, and wash the shells with distilled water. Dry the shells by pressing them against absorbent paper.

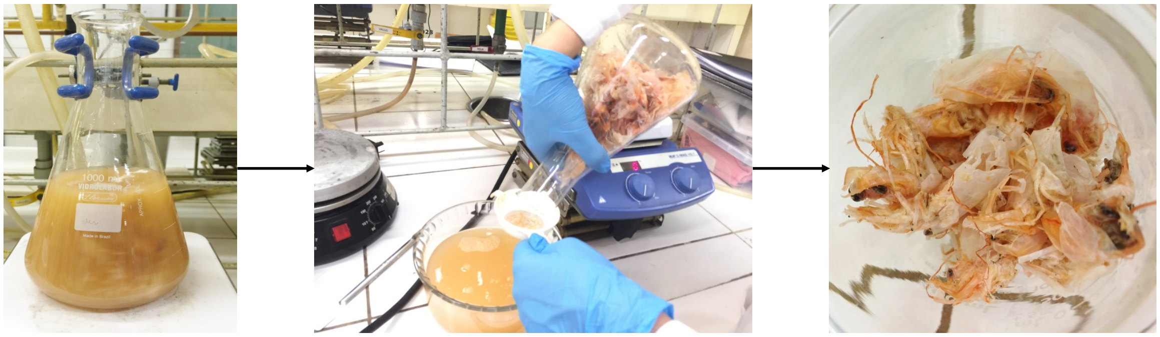

Figure S2. Pictures taken during removal of minerals from shrimp shells.

1.2. Experimental procedure for deproteinization of demineralized shrimp shells. Introduce the demineralized shrimp shells in a 1L round-bottom flask and add 400 mL of an aqueous solution of NaOH 1.0 M. Heat the reaction mixture at reflux during 2h. Filter the solution using a plastic sieve and wash the shells with distilled water. Dry the resulting chitin by pressing it against the absorbent paper.

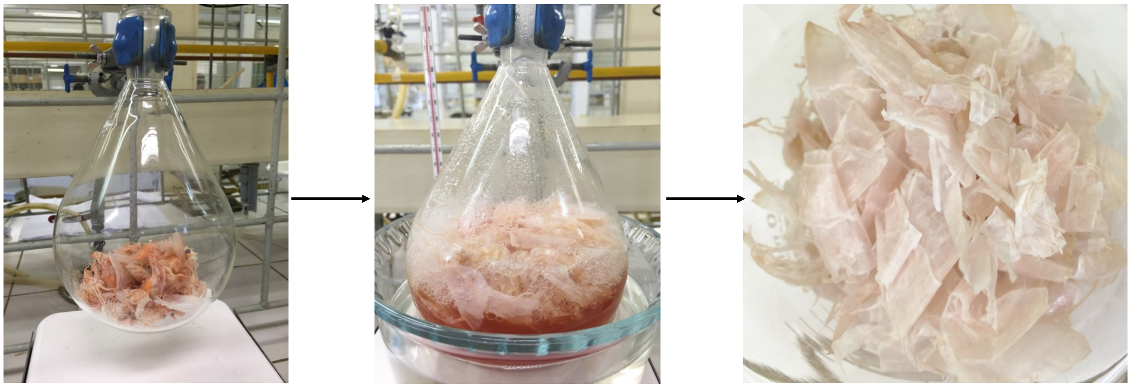

Figure S3. Pictures taken during removal of proteins, pigments and fats from shrimp shells.

1.3. Experimental Procedure for Chitin Hydrolysis to obtain glucosamine hydrochloride. Introduce 25 g of chitin in a 500 mL round-bottom flask and add 80 mL of concentrated HCl (37%). Attach the condenser to the round-bottom flask. Under stirring, heat the solution at 90 ºC for 2h (it is important to keep the temperature below 95ºC, in order to minimize degradation of D-glucosamine). In this step, chitin dissolves and the solution color becomes brownish. To neutralize the HCl vapors released from the reaction mixture, connect one hose to the glass inlet adapter at the top of the condenser, and connect the other hose-end to the gas-washing bottle. Connect another hose to the gas-washing bottle, and plunge the other hose-end into 200 mL of the 10% KOH aqueous solution in an Erlenmeyer (Figure S4). Cool the solution to 60ºC, and add 10 mL of distilled water. Add three spoons of activated charcoal, and keep the solution at 60ºC, under stirring, for 30 min. Filter the solution through paper to obtain a transparent colorless or yellowish solution. Slowly add 100 mL of ethanol and keep the solution in the fridge for several days to allow crystallization of glucosamine hydrochloride. Filter the solution and dry the white crystals under high vacuum.

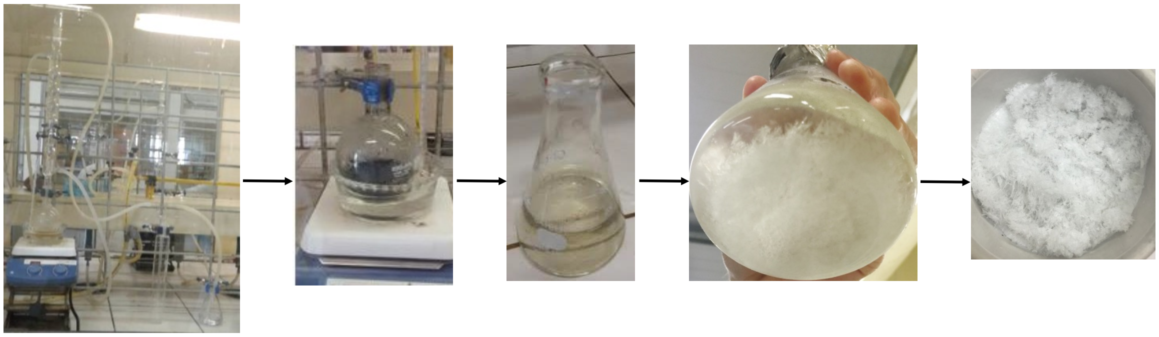

Figure S4. Pictures taken during hydrolysis of chitin and crystallization lead to white crystals of glucosamine hydrochloride.

2. Glucosamine Hydrochloride from Cicada Sloughs

Cicada sloughs. Sloughs from Giant Cicadas (*Quesada gigas*) were collected from trees located on campus (Campinas, São Paulo, Brazil) during October 2015 and October 2016 (Spring). They were used with no previous treatment.

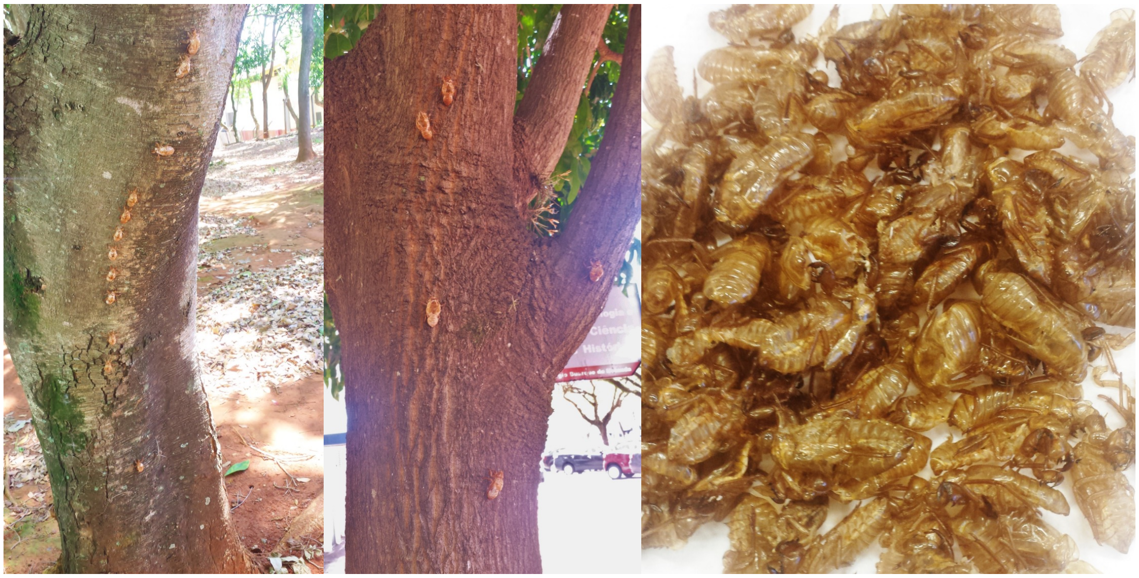

Figure S5. Pictures of the cicada sloughs hanging on trees in our campus (Campinas, São Paulo, Brazil), and after collecting.

2.1. Experimental procedure for demineralization of cicada sloughs.

Introduce 17 g of cicada sloughs in a 1L Erlenmeyer and add 700 mL of an aqueous solution of HCl 0.5 M. Stir the suspension at room temperature, for 3h. Filter the solution using a plastic sieve and wash the residue with distilled water. Dry the sloughs by pressing them against absorbent paper.

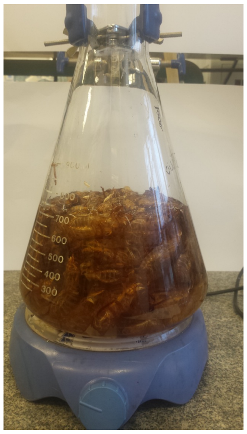

Figure S6. Picture taken during removal of minerals from cicada sloughs.

2.2. Experimental procedure for deproteinization of cicada sloughs. Introduce the demineralized cicada sloughs in a 1L round-bottom and add 400 mL of an aqueous solution of NaOH 1.0 M. Heat the suspension at reflux for 2h. Filter the solution using a plastic sieve and wash the residue with distilled. Dry the resulting chitin by pressing it against absorbent paper.

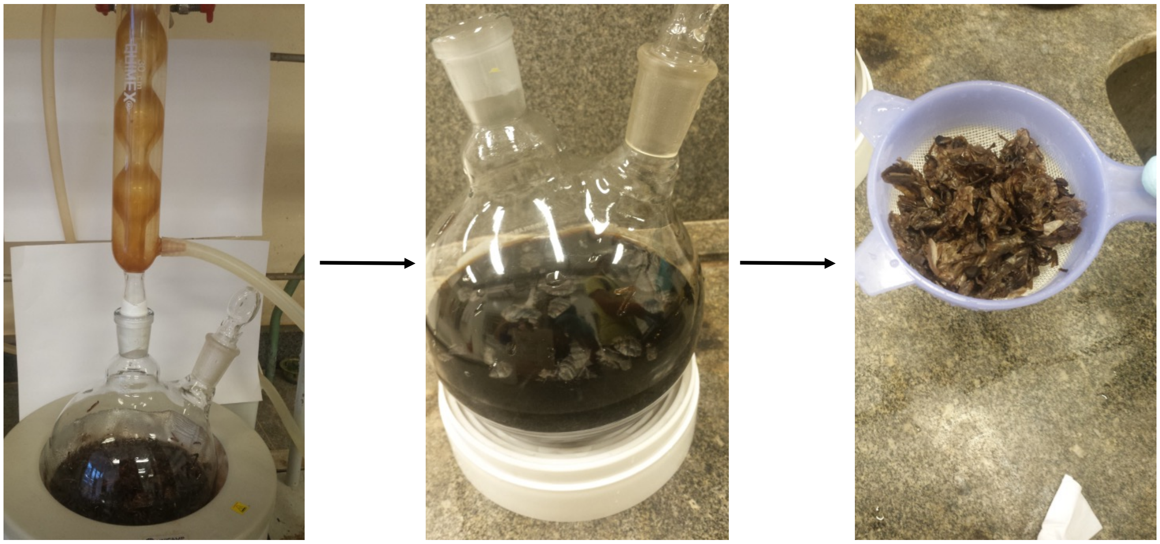

Figure S7. Pictures taken during removal of proteins, pigments and fats from cicada sloughs.

2.3. Experimental procedure for chitin hydrolysis from cicada sloughs. Introduce the chitin obtained in the previous step in a 500 mL round-bottom flask and add 80 mL of concentrated HCl (37%). Attach the condenser to the round-bottom flask. Under stirring, heat the solution at 90 ºC for 2h (it is important to keep the temperature below 95ºC, in

order to minimize degradation of D-glucosamine). In this step, chitin dissolves and the solution color becomes brownish. To neutralize the HCl vapors released from the reaction mixture, connect one hose to the glass inlet adapter at the top of the condenser, and connect the other hose-end to the gas-washing bottle. Connect another hose to the gas-washing bottle, and plunge the other hose-end into 200 mL of the 10% KOH aqueous solution in an Erlenmeyer. Cool the solution to 60ºC and add 10 mL of distilled water. Add activated charcoal, and keep the solution at 60ºC under stirring for 30 min. Filter the solution through paper to obtain a colorless or yellowish solution. Slowly add 100 mL of ethanol and keep the solution in the fridge to allow crystallization of glucosamine hydrochloride. Filter the solution and dry the white crystals under high vacuum.

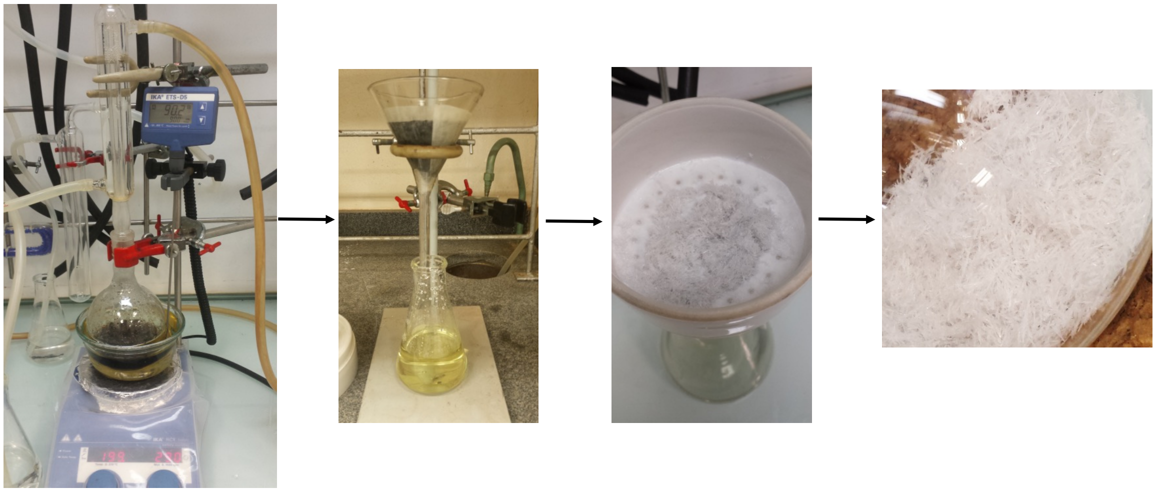

Figure S8. Pictures taken during chitin hydrolysis from cicada sloughs to afford white crystals of glucosamine hydrochloride.

3. Glucosamine Hydrochloride from Cockroaches

Nymph and adult American cockroaches were handpicked in random locations in Campinas, São Paulo, Brazil, and used with no previous treatment.

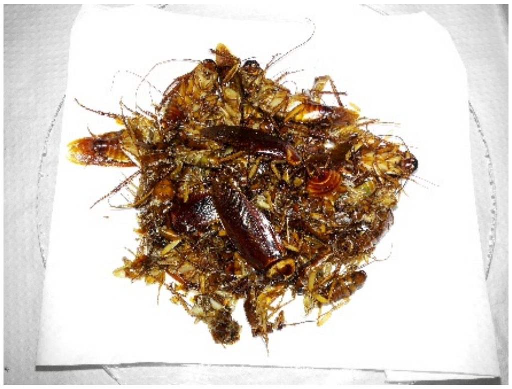

Figure S9. Pictures of the American cockroaches used in our experiment.

3.1. Experimental procedure for demineralization of cockroaches. Introduce 32 g of cockroaches in a 1L Erlenmeyer and add 700 mL of an aqueous solution of HCl 0.5 M. Stir the suspension at room temperature, for 3h. Filter the solution using a plastic sieve, and wash the residue with distilled water. Dry the cockroaches by pressing them against absorbent paper.

3.2. Experimental procedure for deproteinization of cockroaches. Introduce the demineralized cockroaches from the previous step in a 1L round-bottom flask and add 400 mL of an aqueous solution of NaOH 1.0 M. Heat the solution at reflux during 2h. Filter the solution using a plastic sieve, and wash the residue with distilled water. Dry the resulting chitin by pressing it against absorbent paper.

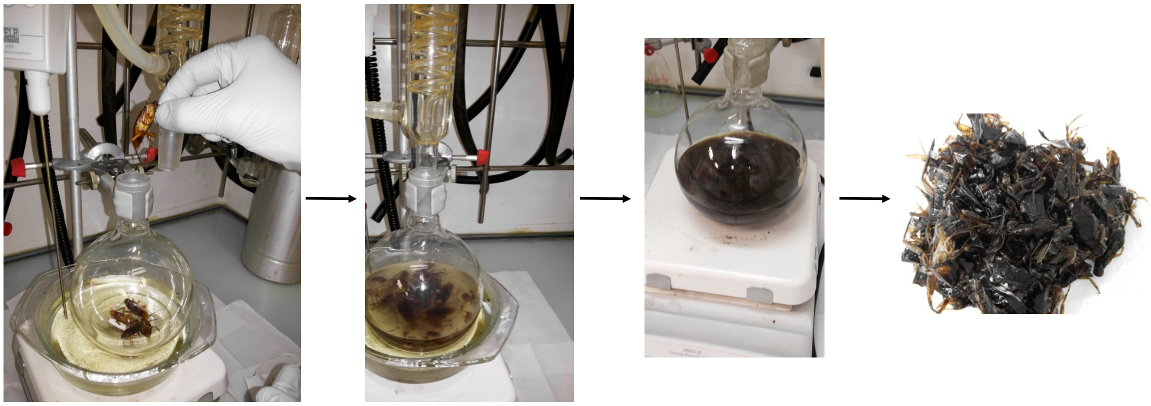

Figure S10. Pictures taken during removal of proteins, pigments and fats from cockroaches.

3.3. Experimental Procedure for chitin hydrolysis from cockroaches. Introduce the **c**hitin obtained in the previous step in a 500 mL round-bottom flask and add 80 mL of concentrated HCl (37%). Attach the condenser to the round-bottom flask. Under stirring, heat the solution at 90 ºC for 2h (it is important to keep the temperature below 95ºC, in order to minimize degradation of D-glucosamine). In this step, chitin dissolves and the solution color becomes brownish. To neutralize the HCl vapors released from the reaction mixture, connect one hose to the glass inlet adapter at the top of the condenser, and connect the other hose-end to the gas-washing bottle. Connect another hose to the gas-washing bottle, and plunge the other hose-end into 200 mL of the 10% KOH aqueous solution in an Erlenmeyer. Cool the solution to 60ºC and add 10 mL of distilled water. Add activated charcoal, and keep the solution at 60ºC under stirring for 30 min. Filter the solution through paper to obtain a colorless or yellowish solution. Slowly add 100 mL of ethanol and keep the solution in the fridge to allow crystallization of glucosamine hydrochloride. Filter the solution and dry the white crystals under high vacuum.

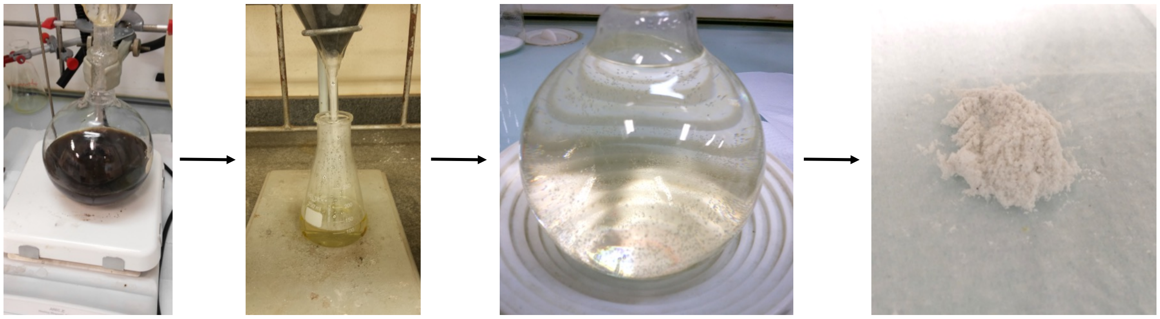

Figure S11. Pictures taken during chitin hydrolysis to obtain white crystals of glucosamine hydrochloride.

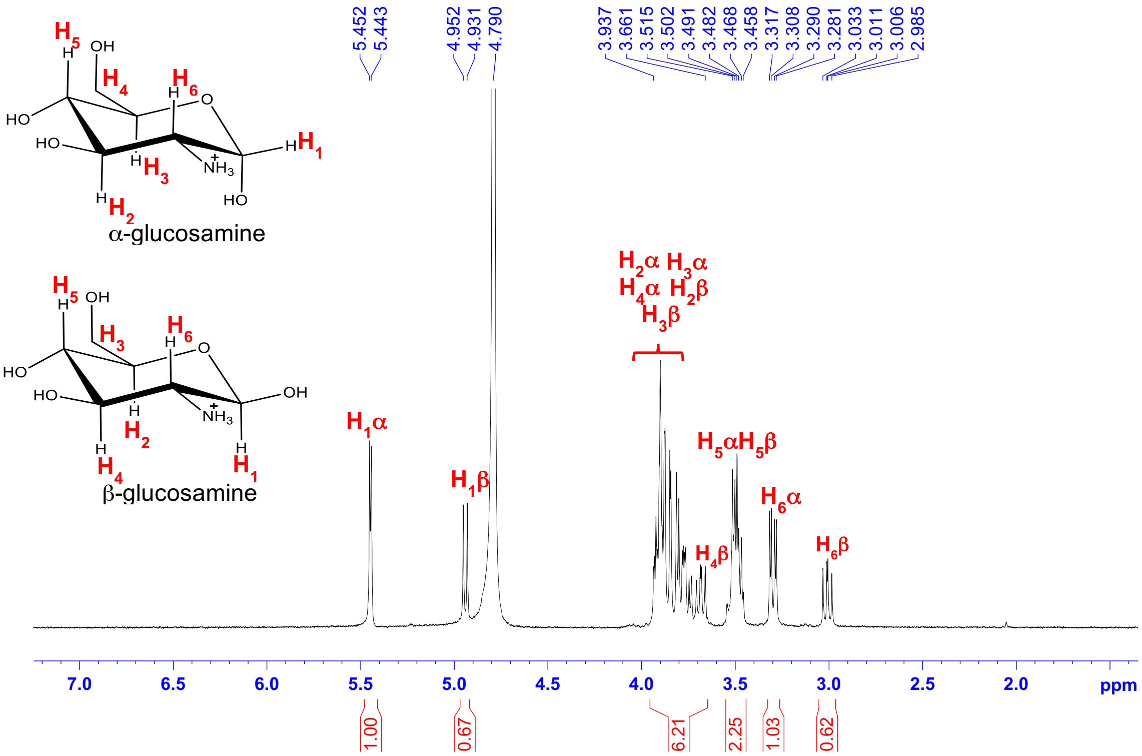

Figure S12. ^{1}H NMR spectrum of glucosamine hydrochloride (400 MHz, D_2O, 25°C).

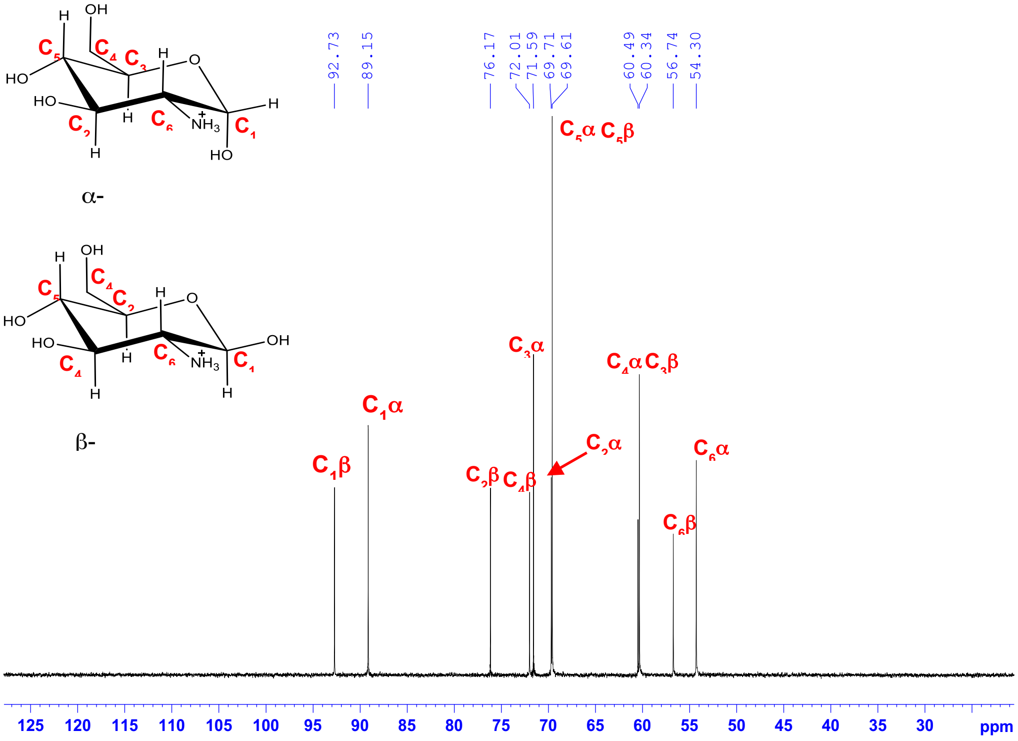

Figure S13. ^{13}C NMR spectrum of glucosamine hydrochloride (100 MHz, D_2O, 25°C).

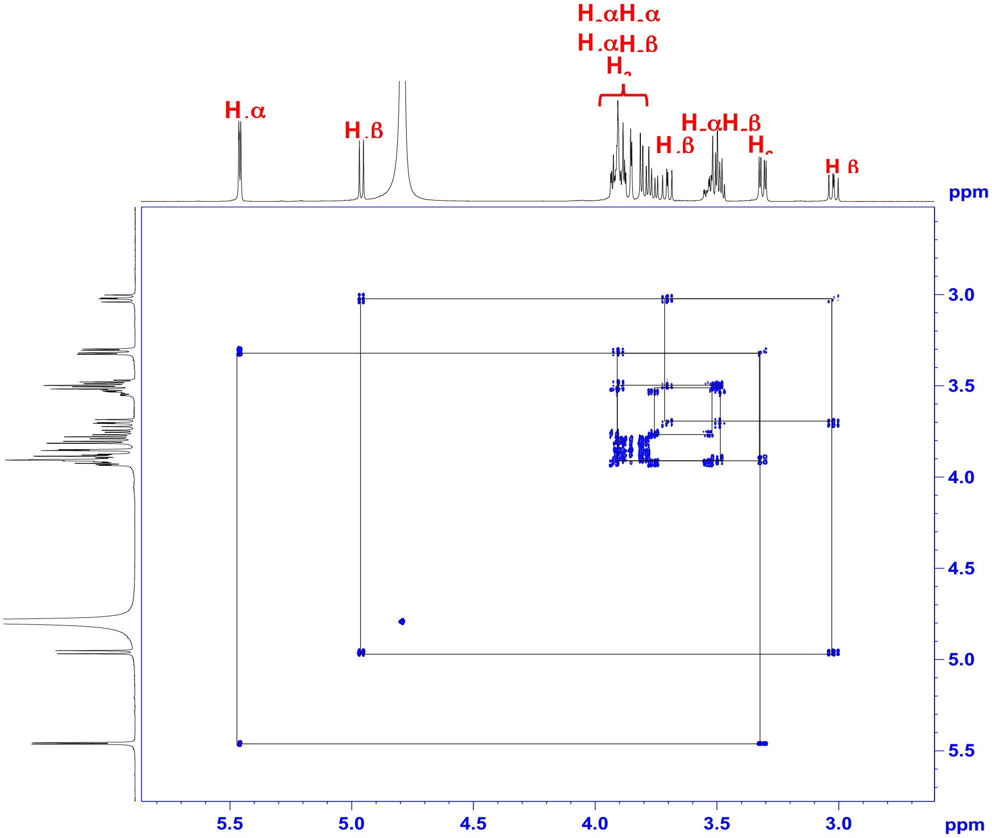

Figure S14. COSY NMR spectrum of glucosamine hydrochloride (400 MHz, D_2O, 25°C).

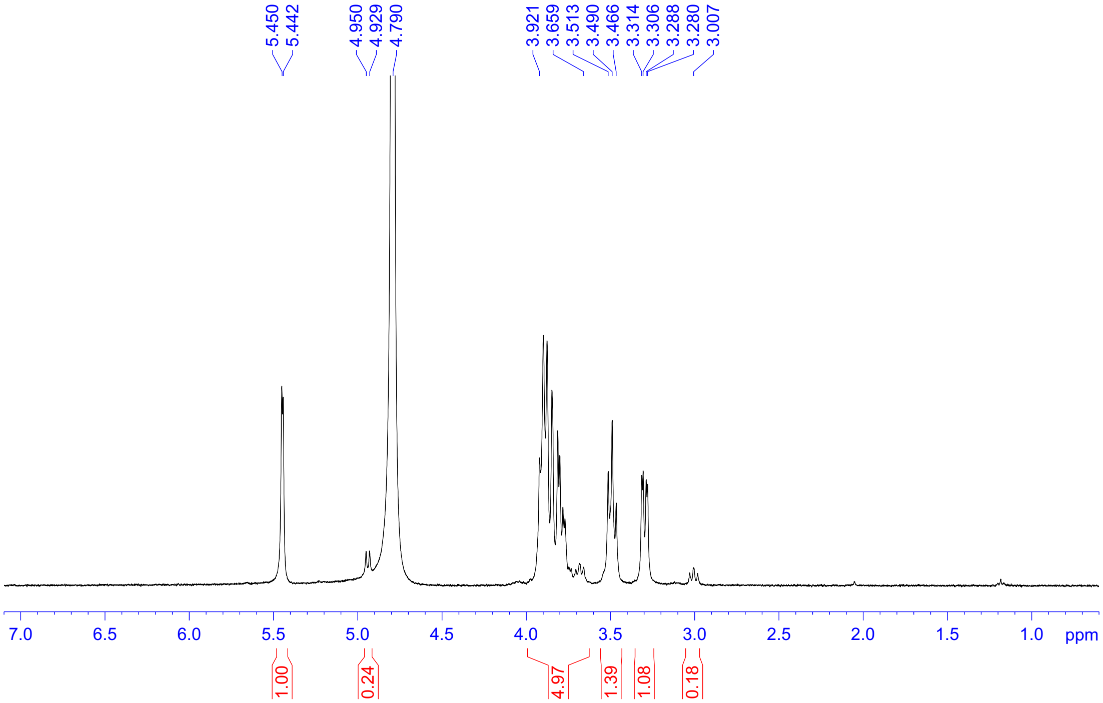

Figure S20. ^{1}H NMR spectrum of glucosamine hydrochloride in D_2O at t=20 min.

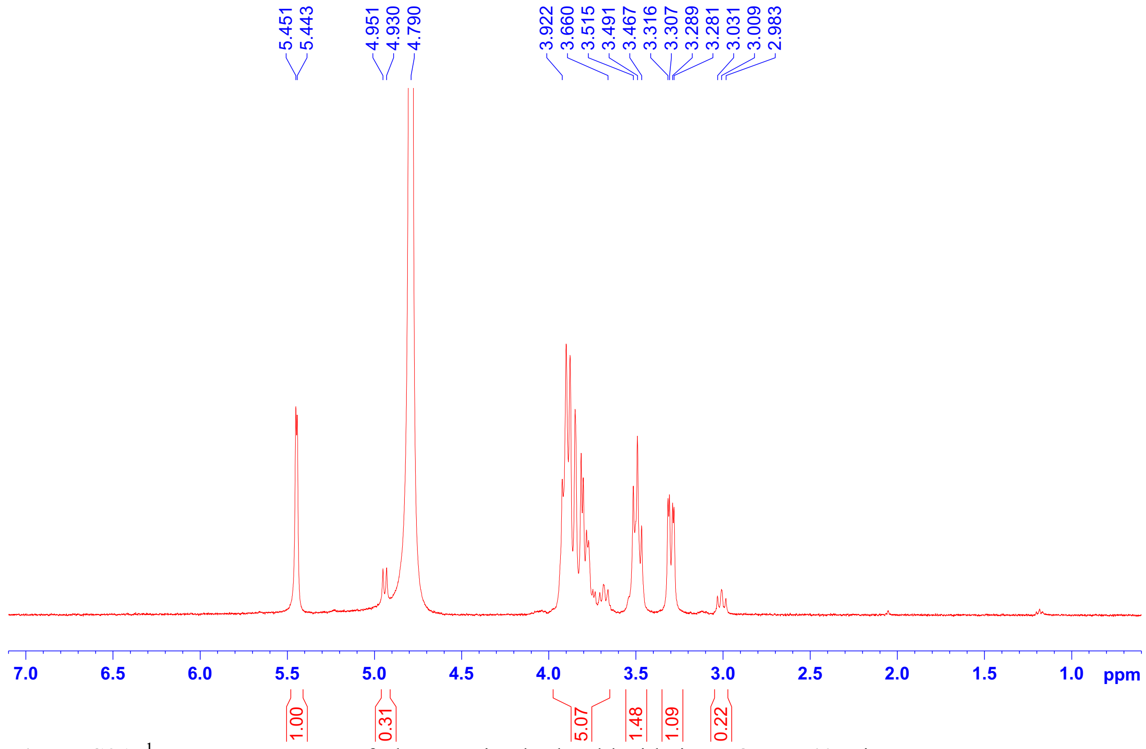

Figure S21. ^{1}H NMR spectrum of glucosamine hydrochloride in D_2O at t=40 min.

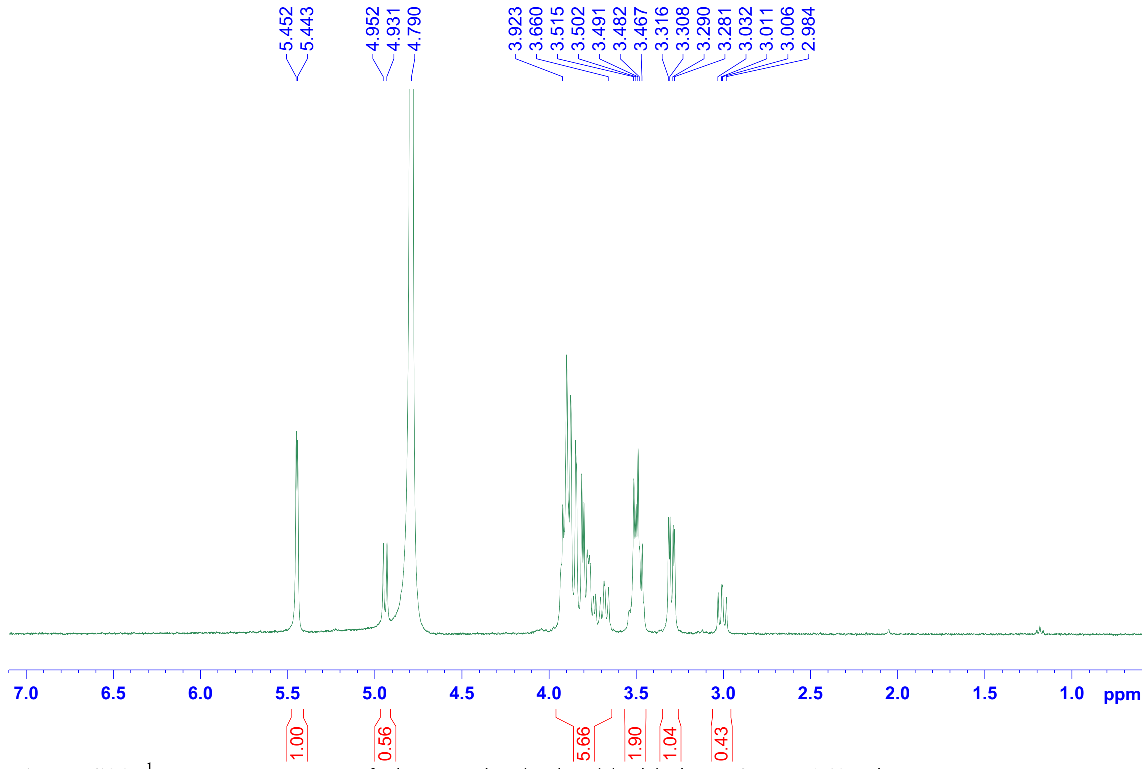

Figure S22. ^{1}H NMR spectrum of glucosamine hydrochloride in D_2O at t=160 min.

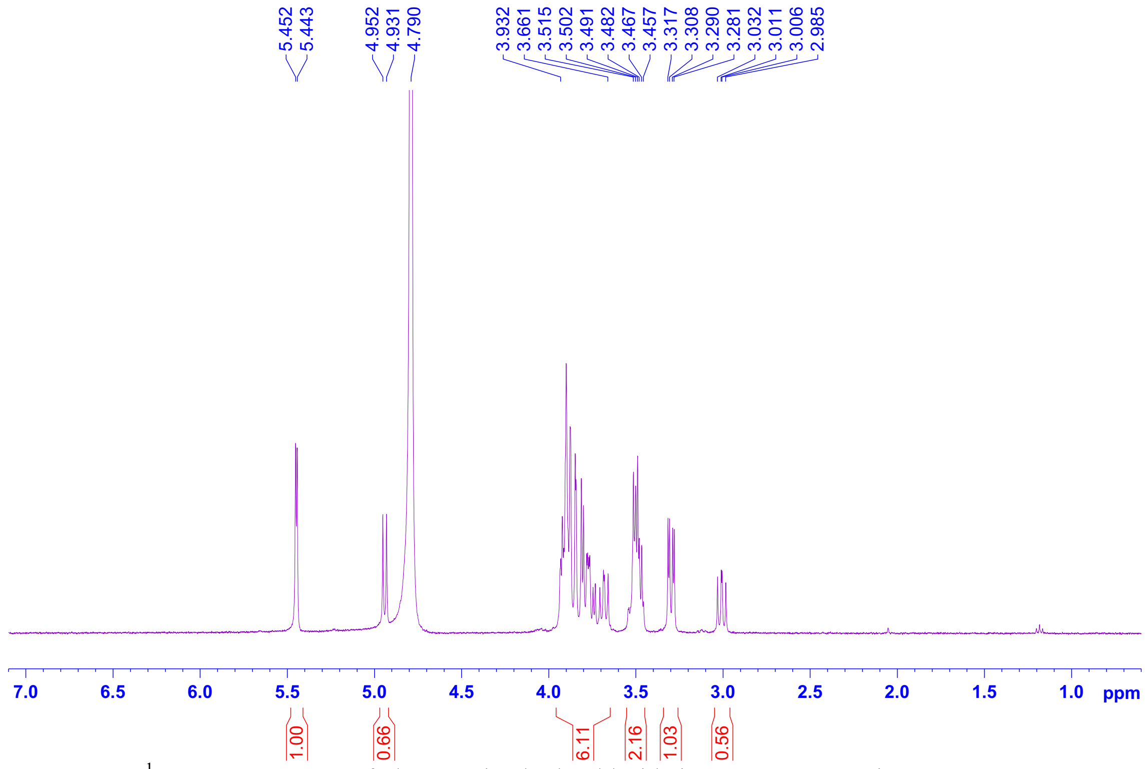

Figure S23. ^{1}H NMR spectrum of glucosamine hydrochloride in D_2O at t=440 min.

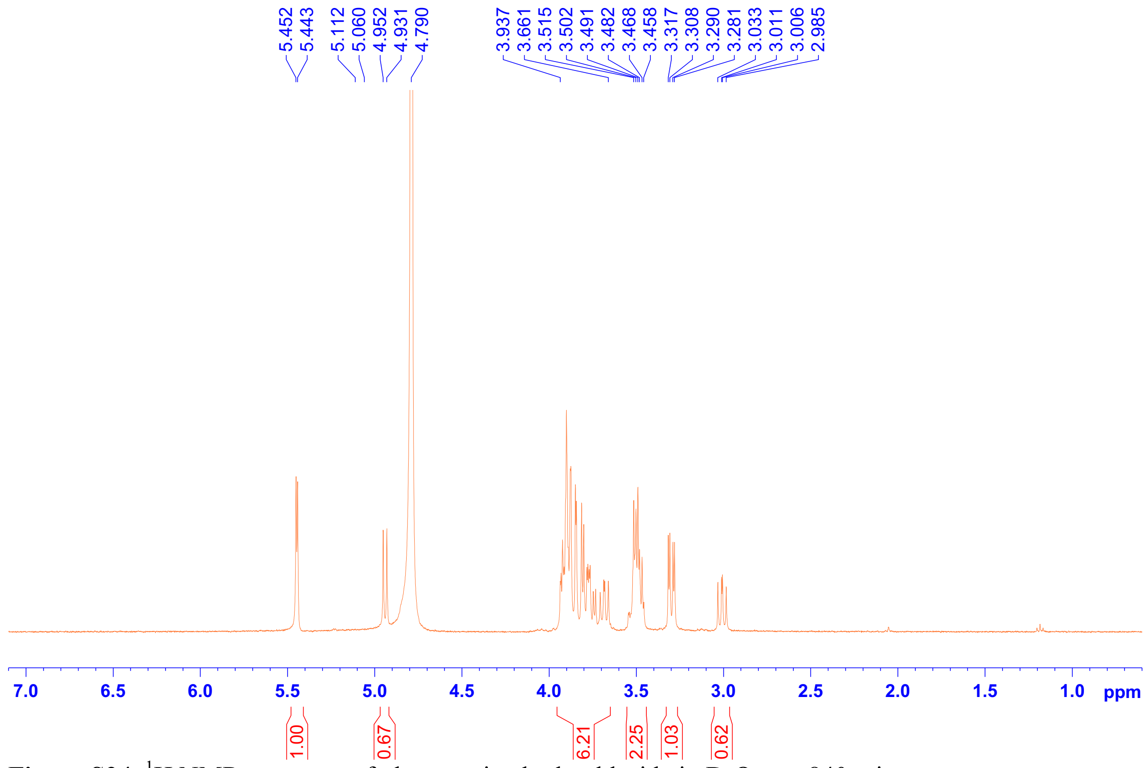

Figure S24. ^{1}H NMR spectrum of glucosamine hydrochloride in D_2O at t=840 min.
